# Herbal Medicine *Maekmundong-Tang* on Patients with Nonspecific Chronic Cough: Study Protocol for a Double-Blind, Randomized Controlled Clinical Trial

**DOI:** 10.3390/ijerph20054164

**Published:** 2023-02-25

**Authors:** Boram Lee, Hyo-Ju Park, So-Young Jung, O-Jin Kwon, Yang-Chun Park, Changsop Yang

**Affiliations:** 1 KM Science Research Division, Korea Institute of Oriental Medicine, Daejeon 34054, Republic of Korea; 2 Clinical Research Coordinating Team, Korea Institute of Oriental Medicine, Daejeon 34054, Republic of Korea; 3 Division of Respiratory Medicine, Department of Internal Medicine, College of Korean Medicine, Daejeon University, Daejeon 34520, Republic of Korea

**Keywords:** chronic cough, *Maekmundong-tang*, *Maimendong-tang*, *Bakumondo-to*, *Saengmaek-san*, herbal medicine, randomized controlled trial

## Abstract

As the treatment of nonspecific chronic cough with conventional medications that treat cough according to the cause is limited, *Maekmundong-tang* (comprising Liriopis seu Ophiopogonis Tuber, Pinelliae Tuber, Oryzae Semen, Zizyphi Fructus, Ginseng Radix, and Glycyrrhizae Radix et Rhizoma) has been used empirically in the clinical setting of East Asian traditional medicine. This study is the first to explore the feasibility, preliminary effect, safety, and cost-effectiveness of *Maekmundong-tang* for nonspecific chronic cough. This study protocol is that of a double-blind, randomized, active-controlled, parallel-group clinical trial for comparing *Maekmundong-tang* with *Saengmaek-san* (comprising Liriopis seu Ophiopogonis Tuber, Ginseng Radix, and Schisandrae Fructus), a Korean national health insurance-covered herbal medicine for cough. A total of 30 nonspecific chronic cough patients will participate and receive the assigned herbal medicine for 6 weeks, and clinical parameters will be assessed at weeks 0 (baseline), 3 (midterm assessment), 6 (primary endpoint), 9, and 24 (follow-up). Study feasibility outcomes, including recruitment, adherence, and completion rates, will be assessed. Preliminary effects on cough severity, frequency, and quality of life will be evaluated using outcome measures, such as the Cough Symptom Score, Cough Visual Analog Scale, and the Leicester Cough Questionnaire. Adverse events and laboratory tests will be monitored for safety evaluation, and exploratory economic evaluations will be conducted. The results will provide evidence of *Maekmundong-tang* in the treatment of nonspecific chronic cough.

## 1. Introduction

The prevalence of cough is about 2.3–18% of the adult population worldwide, and among them, the prevalence of chronic cough, lasting more than 8 weeks in adults, is reported to be about 10–38% [1,2]. Common causes of chronic cough include upper airway cough syndrome, asthma including cough variant asthma, and gastroesophageal reflux disease [2]. In most cases, the cause of chronic cough can be identified and treated if appropriate examination and treatment are performed according to the guidelines and protocols presented in various clinical practice guidelines [3,4]. However, in the case of some chronic coughs, it is difficult to identify the cause and the cough cannot be treated even with these tests and treatments. Recent opinions suggest that these cases should be approached as a different clinical disease [5].

Nonspecific chronic cough is defined as cases where no specific findings are observed in history taking, physical examination, chest X-ray, and pulmonary function test in patients with chronic cough [6]. In conventional medicine, oral antihistamines, inhaled corticosteroids, leukotriene receptor antagonists, and proton pump inhibitors have been suggested empirically for the treatment of nonspecific chronic cough according to symptoms [6]. However, concerns about the side effects during long-term use and the lack of evidence have raised questions about their effectiveness [6,7]. As a result, many patients are seeking complementary and integrative medicine, including herbal medicine, to treat chronic cough [8,9].

*Maekmundong-tang* (*Maimendong-tang* in Chinese, *Bakumondo-to* in Japanese) is an herbal medicine that has long been used in clinical settings for the treatment of nonspecific chronic cough. It consists of Liriopis seu Ophiopogonis tuber, Pinelliae tuber, Oryzae semen, Zizyphi fructus, Ginseng Radix, and Glycyrrhizae Radix et Rhizoma. It was first introduced in Essential Prescriptions from the Golden Cabinet (Jingui Yaolüe), which explains treating cough and discomfort in the throat with qi reflux. While the effects of *Maekmundong-tang* on coughs related to chronic airway diseases and cough hypersensitivity accompanied by chronic cough have been reported by various experimental and clinical studies [10,11,12,13], the evidence is still uncertain. In addition, the effect of *Maekmundong-tang* on chronic cough has not been reported since the time nonspecific chronic cough has been clearly defined. Therefore, in this study, we will confirm the feasibility of large-scale clinical trials based on recruitment, adherence, and completion rates by administering *Maekmundong-tang* to patients with nonspecific chronic cough. In addition, *Saengmaek-san* (*Shengmai-san* in Chinese, *Shomyaku-san* in Japanese; comprising Liriopis seu Ophiopogonis Tuber, Ginseng Radix, and Schisandrae Fructus), the health insurance covered herbal medicine for cough treatment, was set as a control intervention to explore the possibility of *Maekmundong-tang*’s health insurance coverage through a cost-effectiveness analysis.

## 2. Methods

### 2.1. Study Design and Recruitment

**Figure 1:**
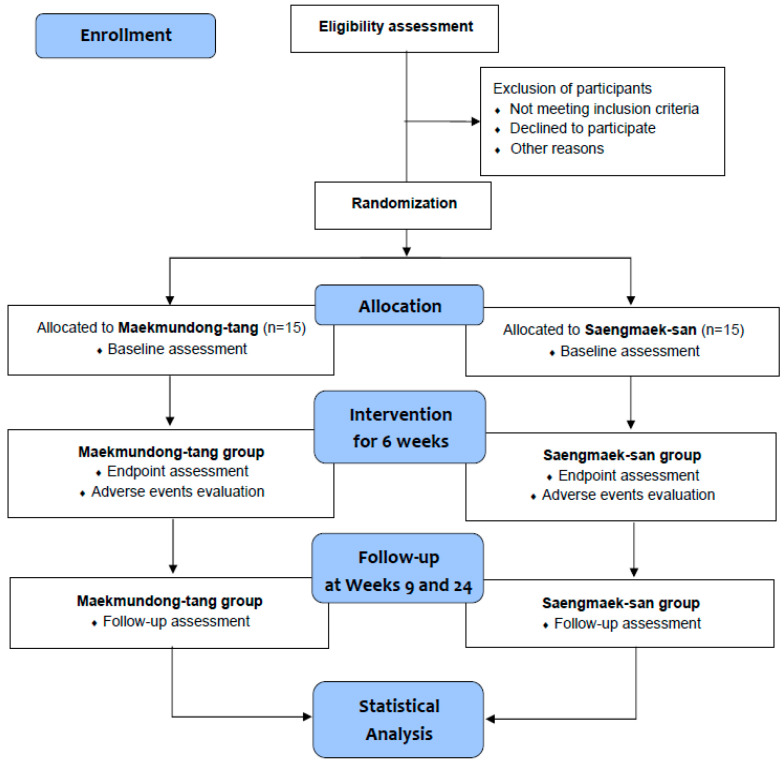
Flow chart of the trial process.

A single-center, double-blind, randomized, active-controlled, parallel-group clinical trial will be conducted at the Daejeon Korean Medicine Hospital of Daejeon University in the Republic of Korea from February 2023 to December 2024. Only those individuals who voluntarily sign the written informed consent form to participate in the clinical trial after receiving a comprehensive explanation from the licensed Korean medicine doctors about the trial will be tested at the screening visit. Participants who satisfy the eligibility criteria will visit the hospital within 7 days to 3 weeks after the screening visit and will be randomly allocated to either the *Maekmundong-tang* group or the *Saengmaek-san* group in a 1:1 ratio. All groups will take investigational products for 6 weeks, and participants will visit the hospital every 3 weeks to undergo clinical evaluation. After taking the drug, a revisit will be performed 3 weeks later (week 9) to evaluate the effect and safety, and a telephone follow-up will be conducted at week 24 to conduct the economic evaluation (Table 1 and Figure 1). In summary, this study has a total of 6 visits (including 1 phone survey), and it will take approximately 6 months. To recruit participants, recruitment documents will be posted on hospital bulletin boards, websites, and applications. Additionally, subway, bus, and newspaper advertisements will also be implemented if recruitment is delayed. All trial processes will be conducted in accordance with the Declaration of Helsinki and Good Clinical Practice Guidelines. In particular, *Maekmundong-tang* has cough as an indication. In the Republic of Korea, a clinical trial is exempted from investigational new drug application if it targets a drug already approved by the Korean Ministry of Food and Drug Safety and is conducted within the scope of the indication permitted [14]. Therefore, pre-clinical data such as toxicity data are unnecessary for this type of clinical trial.

### 2.2. Inclusion Criteria

(1) adults between the ages of 19 and 75

(2) those who have a cough lasting more than 8 weeks

(3) those who have signed an institutional review board (IRB)-approved written informed consent form to participate in this study voluntarily

### 2.3. Exclusion Criteria

(1) those who have been diagnosed with other causes of chronic coughs, such as chronic respiratory diseases (chronic obstructive pulmonary disease, bronchial asthma, bronchiectasis, interstitial lung disease, pulmonary tuberculosis, etc.), gastroesophageal reflux disease, chronic sinusitis, and allergic rhinitis within the last 2 years

(2) those who have the following symptoms that can be presumed to cause cough: wheezing, chest tightness, dyspnea, runny nose, nasal congestion, sneezing, postnasal drip, abnormal findings in the ear canal, heartburn, reflux, fever, and hemoptysis

(3) those with abnormal findings on a chest X-ray that may cause cough 

(4) those with abnormal findings in the pulmonary function tests

(5) those with wheezing, rales, postnasal drip, or cobblestone throat during the physical examination

(6) those who have been diagnosed with an acute respiratory disease within the last 4 weeks

(7) those who are current smokers, quit smoking within 3 months, or have a history of excessive smoking in the past 30 pack years or more

(8) those who are taking angiotensin-converting enzyme (ACE) inhibitors or dipeptidyl peptidase-4 (DPP-4) inhibitors or have taken them within the last 4 weeks

(9) those who have taken antitussives, expectorants, steroids, anti-leukotrienes, anticholinergics, long-acting beta 2 agonists (LABA), oral antihistamines, or any herbal medicine within the past 2 weeks

(10) those who have been diagnosed with a malignant tumor within the last 5 years

(11) those with severe liver or renal disease (aspartate aminotransferase (AST) or alanine aminotransferase (ALT) levels ≥ 3 times the upper limit of normal or creatinine levels ≥ 2 times the upper limit of normal) 

(12) those with a history of serious alcohol or drug abuse 

(13) women of childbearing age who are pregnant or lactating or who do not agree to use an effective method of contraception during the trial period

(14) those with genetic problems such as galactose intolerance, Lapp lactase deficiency, or glucose-galactose malabsorption

(15) those with known hypersensitivity to the investigational products 

(16) those who had taken other investigational products within 3 months 

(17) those who have not recorded a cough diary for at least 7 days immediately prior to the baseline visit

(18) those with average daytime cough symptom score (CSS) < 2 points and night-time CSS < 1 point according to the cough diary recorded for the 7 days immediately before the baseline visit

(19) those who are judged to be inappropriate for participating in this study by the investigators

### 2.4. Randomization and Blinding

The randomization sequence will be generated by block randomization method (mixing block sizes of two and four) without stratification. The allocation ratio for each group is 1:1. The randomization sequence will be generated using the statistical program SAS^®^ Version 9.4 (SAS Institute Inc., Cary, NC, USA) by a medical statistician, independent of the interventions and evaluations in this clinical trial. Participants who meet all the eligibility criteria will be allocated from the minimum available number to each group. 

In this clinical trial, all participants, investigators, and outcome assessors are blinded. Each participant’s group information according to the randomization order will be put in an opaque sealed envelope. It will not be disclosed until the end of the clinical trial, except when it is unavoidable to view group information due to the occurrence of a serious adverse event.

### 2.5. Interventions

All investigational drugs were manufactured by Kyungbang New Pharmaceutical Co., Ltd. (Incheon, Republic of Korea) in compliance with the 2021 Korean Good Manufacturing Practice guidelines. All participants will be allocated to the *Maekmundong-tang* or *Saengmaek-san* groups. Each 2.5-g serving of *Maekmundong-tang* granule (Yunpye-tang extract granule, Maekmundong-tang; product code: 200005808) comprises grayish-brown granules that consist of 1250 mg *Maekmundong-tang* dry extract, 625 mg corn starch, and 625 mg lactose hydrate. The raw materials and composition of *Maekmundong-tang* dry extract 1250 mg are as follows: Liriopis seu Ophiopogonis Tuber 3.33 g, Pinelliae Tuber 1.67 g, Oryzae Semen 3.33 g, Zizyphi Fructus 1.00 g, Ginseng Radix 0.67 g, and Glycyrrhizae Radix et Rhizoma 0.67 g. Similarly, each 1.41 g serving of *Saengmaek-san* powder (Kyungbang *Saengmaek-san*, mixed single extract; product code: 200005617), the active control drug, is a brown powder that consists of 0.75 g Liriopis seu Ophiopogonis Tuber extract, 0.30 g Ginseng Radix extract, and 0.36 g Schisandrae Fructus extract. The investigator will instruct all participants to orally consume 1 pack of the formulation 3 times a day, before or between meals, for 6 weeks. Each of the herbal medicine was produced simultaneously in one batch and therefore has the same batch number. Their expiration date is 36 months from the date of manufacture. They should be stored in airtight containers at room temperature (1–30 °C). 

To improve the compliance of the participants of the clinical trial, the investigator will check the participant’s investigational product intake during the administration period and record details of compliance in the case report form (CRF); participants with less than 70% medication compliance at Week 6 will be dropped out.

The investigator will provide a multidimensional behavioral therapy (speech pathology therapy) guideline, which is a non-pharmacological treatment for cough, to all participants and educate the participants about this therapy [15]. This includes the creation of awareness that repetitive coughing is not beneficial and that coughing can be controlled by oneself through the suppression of the cough reflex that is induced by stimuli by swallowing saliva or water, or breathing with pursed lips when about to cough [15]. In addition, the education includes the vocal hygiene method, which ensures the supply of moisture to reduce laryngeal irritation, and psychological education counseling therapy, which helps patients recognize that chronic cough is difficult to treat and thereby set realistic treatment goals [15].

During the clinical trial period, new intake of any prescription drugs and over-the-counter medicines, except for investigational products and drugs allowed at screening visit, should be avoided. In particular, the trial should be discontinued if the following medications or treatments are required due to the participants’ circumstances or clinical condition: ACE inhibitor, DPP-4 inhibitor, antitussive, expectorants, steroid, antileukotrienes, anticholinergic, LABA, oral antihistamines, traditional Korean treatment (e.g., herbal medicine, acupuncture, etc.) to improve chronic cough, and other drugs or treatments that, in the investigator’s judgment, may affect the results of the clinical trial. 

At any time during the trial, the participants can voluntarily withdraw their participation for reasons such as dissatisfaction with the treatment effect. If participants are no longer able to participate in the trial due to adverse events or intercurrent diseases during the trial or have received the contraindicated drugs, which may affect the research outcomes, the participants will be dropped from the study at the investigators’ discretion. 

### 2.6. Outcome Measures

#### 2.6.1. Study Feasibility Outcomes

As the primary purpose of this clinical trial is to evaluate the feasibility of a large-scale herbal-medicine clinical trial for nonspecific chronic cough, the following eight feasibility outcomes will be assessed in consultation with specialists in respiratory medicine by referring to the Consolidated Standards of Reporting Trials statement for feasibility trials and similar studies [16,17,18]: (1) Recruitment rate of the clinical trial: the percentage of the number of enrolled participants to the total number of participants screened for eligibility during the clinical trial period; (2) Total participant recruitment period of the clinical trial: the time elapsed between the first and last participant’s screening visit (to estimate the recruitment period of the future confirmatory clinical trial); (3) The average number of enrolled participants per month during the clinical trial recruitment period; (4) Average compliance of participants with investigational products: the average percentage of individual participants’ actual use of investigational drugs during the clinical trial period as opposed to the intended use of those drugs; (5) Clinical trial adherence rate: the percentage of participants who take 70% or more of all investigational products compared to the total number of participants enrolled in the clinical trial; (6) Average cough diary record compliance of participants: the average percentage of the number of days in the cough diary actually logged by individual participants during the clinical trial period compared to the intended number of days; (7) Completion rate of clinical trial: the percentage of participants who complete the trial without dropout, including follow-up periods of 9 and 24 weeks, respectively, to the total number of enrolled participants in the clinical trial; and (8) Dropout rate of clinical trial: the percentage of participants who drop out before the end of the clinical trial to the total number of enrolled participants.

#### 2.6.2. Effectiveness Outcomes

The primary effectiveness outcome is the CSS score at Week 6. The CSS is a scale for the subjective assessment of cough severity and frequency that is reported by the patient during the daytime (08:00–20:00) and nighttime (20:00–08:00), and each subdomain of the scale is assigned 6 points, from 0 to 5. The total score is calculated as the sum of the daytime and nighttime scores (0–10 points) [19]. The validity and replicability of the Korean version of CSS have been verified [20]. Until Week 9, participants will be instructed to self-evaluate the CSS twice a day and record the score in a cough diary. During the telephonic follow-up survey (Week 24), participants will be asked to respond on their cough symptoms during the daytime and nighttime of the previous day. For statistical analysis, the average value (total, daytime, and nighttime scores) of the CSS since the previous visit that has been recorded in the cough diary and will be collected at each visit (excluding the baseline visit) will be used, and the average value of the previous 7 days will be used for determining the status at the baseline visit. The investigator will educate the participants on how to write a cough diary at every visit and attempt to minimize omissions in records. If less than 70% of the cough diary distributed at each visit has been completed, the participant will be dropped out.

The secondary outcome measures include the CSS (at weeks 3, 9, and 24), cough visual analog scale (VAS), the Leicester Cough Questionnaire (LCQ), the Hull Airway Reflux Questionnaire (HARQ), Integrative Medicine Outcome Scale (IMOS), Integrative Medicine Patient Satisfaction Scale (IMPSS), a five-level version of the EuroQol Five-dimensional Descriptive System (EQ-5D-5L), the EuroQol visual analog scale (EQ-VAS), yin deficiency score, and serum biomarkers.

For the cough VAS assessment, participants will self-evaluate their subjective discomfort based on cough severity, frequency, and urge to cough on a scale of 0 (no discomfort from coughing) to 100 (the highest discomfort imaginable) for the past 3 weeks [21]. This assessment will be undertaken at every visit, except for the screening visit, as well as during the telephonic follow-up survey (at Week 24), and will be investigated through a numerical rating scale (NRS) score from 0 to 100. The LCQ is a 19-item questionnaire designed to measure the quality of life of patients with cough and is divided into physical, mental, and social categories [22]. Each item is checked on a scale of 1 to 7, with higher scores indicating better health. The Korean version of the LCQ has been verified for validity and reliability [23]. The HARQ is a self-report questionnaire that measures cough hypersensitivity, and each of 14 questions is evaluated on a scale of 0 to 5. For normal individuals, the average score is 4 points, and the upper limit of the normal range is 13 points [24]. The IMOS is a tool for the investigator or participant to evaluate the degree of posttreatment improvement [25], and has been set as the investigator’s evaluation tool in this clinical trial. The IMPSS is an evaluation tool for ascertaining the participant’s treatment satisfaction [25]. Each tool is evaluated on a scale of 1–5 points. The EQ-5D-5L is a self-report questionnaire that is used to evaluate the quality of life, based on a 5-point scale over five areas: mobility, self-care, usual activities, pain/discomfort, and anxiety/depression [26]. The EQ-VAS asks participants to rate their own health from 0 (the worst imaginable health status) to 100 (the best imaginable health status) [26]. In case of telephonic follow-up (at Week 24), the EQ-VAS will be investigated through a 0 to 100 NRS. The Yin Deficiency Questionnaire is a pattern identification questionnaire with 27 questions, and the reliability and validity of this questionnaire have been established. Each question is evaluated on a 7-point Likert scale, of which points 1–4 are converted to 0 points and points 5–7 are converted to 1 point for calculation. The cutoff score for a yin deficiency diagnosis is 10 points [27]. The serum biomarkers include eosinophils, total immunoglobulin E (IgE), and plasma fibrinogen, which will be evaluated at the screening visit and at Week 6. In particular, during the telephonic follow-up survey (at Week 24), CSS, cough VAS, EQ-5D-5L/EQ-VAS, and cost questionnaires will be surveyed through a phone call.

#### 2.6.3. Safety Outcomes

To evaluate safety both before and after the administration of the investigational drugs, liver function (AST and ALT) and kidney function (blood urea nitrogen [BUN] and creatinine) tests will be conducted at the screening visit and at Week 6. In addition, the occurrence and frequency of adverse events will be evaluated at each visit through physical examination conducted by the investigator and through the participant’s self-report; moreover, the vital signs (blood pressure, pulse rate, and body temperature) will be measured at each visit and will be used as safety outcomes.

### 2.7. Sample-Size Calculation

This is the first exploratory clinical trial to evaluate the feasibility and preliminary effect of *Maekmundong-tang* administration in patients with nonspecific chronic cough. Therefore, there are no prior literature references for sample size calculation. Referring to a study that recommended a minimum of 12 people per group in a preliminary study in the field of life sciences [28], a total of 24 participants, with 12 participants per group, are needed. Considering a dropout rate of 20%, a total of 30 participants, 15 in each group, were specified as the target sample size.

### 2.8. Statistical Analysis

The statistical analysis of this clinical trial, in principle, will use full analysis set (FAS) analysis as the main analytical method; if necessary, a per protocol set (PPS) analysis will be additionally conducted. The FAS analysis group is defined as comprising participants with data measured for the outcome measures at least once after being randomly allocated, and all data obtained therefrom are included in the analysis. The PPS analysis comprises only participants included in the FAS analysis who have completed the entire process as specified in the protocol and have no significant violations that affect the trial results. The safety analysis includes all the data obtained from the participants who received the investigational product at least once. 

All statistical analysis is based on a two-sided test, and the significance level is set at 0.05. For continuous data, the mean and confidence interval are presented, whereas for categorical data, the frequency and percentage are presented. Statistics on the demographic and socioeconomic characteristics are presented for each group. Depending on normality, continuous data are analyzed using the independent *t*-test or Wilcoxon rank sum test, whereas categorical data are analyzed using the chi-square or Fisher’s exact test. For analyzing the effectiveness outcomes, the mixed-effect model repeated-measures method will be used, with each group and visit as the fixed factor and the participants as the random factor. If necessary, variables that show statistical differences in demographics or variables that can affect chronic cough will be set as fixed factors and analyzed. Intragroup differences in values before and after treatment will be analyzed, based on normality, with the Student’s paired *t*-test or Wilcoxon signed rank test. The IMOS and IMPSS scores are analyzed using Fisher’s exact test. The repeated-measures analysis of variance method is used to compare the difference in trend change between groups for the effectiveness outcomes, and Dunnett’s procedure is used for correction of the multiple-comparison analysis. If necessary, subgroup analysis will be performed by categorizing the participants according to their baseline characteristics (chronic cough pattern identification [29], presence of yin deficiency [27], sex, age, etc.). For the analysis of safety outcomes, the chi-square or Fisher’s exact test will be performed to compare the number of adverse events in the study groups. In addition, AST, ALT, BUN, creatinine, and vital signs will be analyzed using a paired *t*-test or Wilcoxon signed rank test to determine differences from before to after treatment. No interim analysis has been planned. All statistical analyses will be performed by a statistician, independently of trial procedure, by using the statistical program SAS^®^ Version 9.4 (SAS Institute Inc., Cary, NC, USA). 

### 2.9. Economic Evaluation

Economic evaluation will be conducted along with the randomized controlled trial (RCT) for feasibility, effectiveness, and safety. The cost-effectiveness of *Maekmundong-tang* will be estimated compared to *Saengmaek-san*, in regard to the healthcare system and societal perspective.

Medical, non-medical (transportation, time, and nursing care costs), and productivity loss costs due to participants’ chronic cough during the trial period will be estimated using the cost questionnaires, institutional data, and if necessary, data from Statistics Korea. Especially, productivity loss will be assessed using the Work Productivity and Activity Impairment Questionnaire: General Health V2.0 [30,31]. With regard to the effectiveness data, trial outcome measures, including EQ-5D-5L/EQ-VAS, CSS, and cough VAS, will be determined and, thereby, the incremental cost–utility ratio and the incremental cost-effectiveness ratio between *Maekmundong-tang* and *Saengmaek-san* will be calculated. 

The analysis period is based on the clinical trial period, and for participants who drop out, data will be collected until the end of the administration of the investigational product for each participant. However, if an individual participant drops out due to an adverse event or intercurrent disease during the clinical trial, or due to aggravation of cough symptoms, a telephonic follow-up will be conducted at Week 24 with the consent of the participants and without being excluded from the data collection of the economic evaluation study. If necessary, one-way sensitivity analysis will be performed for possible variables, a tornado diagram will be presented, and a probability sensitivity analysis will be performed using the distribution and representative values of variables. In addition, if necessary, subgroup analysis can be performed according to the subgroup analysis plan of the trial. If the full yield cost and effectiveness outcomes of interventions during the trial period cannot be observed, a decision-analytic model will be developed to conduct a long-term evaluation. 

### 2.10. Data Collection, Management, and Monitoring

In this clinical trial, participants will visit the institution a total of 6 times (including the Week 24 telephonic survey), and the investigator will collect data from the participants at each visit. The Korea Institute of Oriental Medicine (KIOM), a research fund support institution, produced a standard operating procedure that was distributed to those who are related directly to the study to standardize data and improve the quality of clinical trials. Based on this, education for researchers was conducted, and standardization of data collection and evaluation methods for clinical trials was sought. 

For data collection and management, an online CRF iCReaT (Osong, Republic of Korea) will be used, and it will be managed by a data manager (DM) from the KIOM. Access to clinical trial data will be allowed only to those who are involved directly in the study to protect confidentiality. The trial data will be entered by one researcher at the institution, and it will go through two verification processes by a clinical research associate (CRA) and the DM to improve data quality. 

For data monitoring, the CRA will monitor the clinical trial through regular visits to institutions, evaluate the trial progress, including study progress and adverse events, and confirm whether investigators are fulfilling their obligations according to the trial protocol and regulations. Currently, there are no planned audits. 

### 2.11. Ethics Approval

The protocol has been approved by the IRB of Daejeon Korean Medicine Hospital of Daejeon University (DJDSKH-22-DR-18). The trial protocol has been registered at the Clinical Research Information Service (CRIS; registration number: KCT0007990). Any modifications to the protocol will be approved by the IRB and documented in the CRIS. The results of this trial will be disseminated after peer review and publication.

## 3. Discussion

This is the protocol for a single-center, double-blind, randomized, active-controlled, parallel-group clinical trial to assess the study feasibility, preliminary effectiveness, safety, and cost-effectiveness of *Maekmundong-tang* for patients with nonspecific chronic cough. *Maekmundong-tang* has been approved as an over-the-counter drug by the Korean Ministry of Food and Drug Safety for indications of “cough with persistent phlegm, bronchitis, and bronchial asthma” in the Republic of Korea. In addition, *Maekmundong-tang* and *Saengmaek-san* are herbal medicines available in China, Japan, and Korea. Therefore, the results of this clinical trial can have international significance.

Especially, nonspecific chronic cough has been recently defined as prolonged cough in the absence of any symptoms, signs, history, or laboratory findings that indicate a specific diagnosis for chronic cough in the clinical practice guideline [6]. In conventional medicine, medications are administered according to the cause of chronic cough. However, various causative factors may be involved in chronic cough, and although the prevalence has not been reported through epidemiological studies to our knowledge, there are some cases of nonspecific chronic cough wherein the cause is difficult to identify clearly in the clinical setting. Due to the combination of these reasons, dissatisfaction with the treatment effect of conventional medicine is reportedly high [32].

Herbal medicine is characterized by a multicomponent, multitarget formulation [33]. Therefore, it is promising for treating chronic cough with unknown or intractable causes. *Maekmundong-tang* is frequently used for the empirical treatment of nonspecific chronic cough in the East Asian traditional medicine clinical setting. Although the effects of *Maekmundong-tang* on cough related to chronic airway diseases and cough hypersensitivity accompanied by chronic cough have been reported through various experimental and clinical studies [10,11,12,13], robust evidence is still lacking. In addition, although a clinical trial comparing the effect of *Maekmundong-tang* with a placebo control group in patients with chronic dry cough was conducted in the Republic of Korea [34], it did not rule out common causes of chronic cough such as the upper airway cough syndrome. Furthermore, the effect of *Maekmundong-tang* on chronic cough has not been reported since the time nonspecific chronic cough was clearly defined in a recent clinical practice guideline [6]. Therefore, in this exploratory clinical trial of *Maekmundong-tang* in patients with nonspecific chronic cough, the feasibility of large-scale clinical trials, such as the recruitment, adherence, and completion rates, will be confirmed. In addition, the existing health insurance-covered herbal medicine was used in the control group to explore the possibility of medical insurance benefits for *Maekmundong-tang* through cost-effectiveness analysis. 

For the control group intervention, *Saengmaek-san*, for which cough is an indication, and which has been actively studied for chronic cough patients through clinical studies [35,36,37], has been specified. In particular, as nonspecific chronic cough is mainly characterized by dry cough [6,35], *Saengmaek-san*, which has been used for dry cough because of its pulmonary mucolytic effect to suppress cough [38], has been specified as the control intervention based on consultation with experts in respiratory medicine. Through this, we plan to not only explore the preliminary effects, safety, and cost-effectiveness of *Maekmundong-tang* but also observe the clinical effects before and after administration of *Maekmundong-tang* and *Saengmaek-san* for nonspecific chronic cough.

As this study aims to evaluate the feasibility of a large-scale study of *Maekmundong-tang* for nonspecific chronic cough, various feasibility outcomes, including the recruitment, adherence, and completion rates, were set as study feasibility measures by referring to the Consolidated Standards of Reporting Trials (CONSORT) 2010 statement for randomized pilot and feasibility trials [16]. We will evaluate the effects of *Maekmundong-tang* and *Saengmaek-san* on cough severity, frequency, and quality of life through outcome measures, including CSS, cough VAS, and LCQ. In addition, as *Maekmundong-tang* is widely used for paroxysmal cough caused by bronchial hypersensitivity in clinical practice [9], symptoms related to cough hypersensitivity will be explored using HARQ. *Maekmundong-tang* might normalize the production and gene expression of airway mucin that is observed in various respiratory diseases and is accompanied by yin deficiency [39]. Therefore, we will examine the change of yin deficiency using the yin deficiency pattern-identification questionnaire. In addition to the self-reported questionnaire, we will measure changes in serum biomarkers, including eosinophils, total IgE, and plasma fibrinogen [40], to determined the underlying mechanisms of *Maekmundong-tang* and *Saengmaek-san* action for improving cough.

According to a systematic review summarizing the effects of *Maekmundong-tang* on patients with cough, 4 of the 9 included RCTs reported adverse events [9]. Among them, rash (1 case) and elevation of alkaline phosphatase (2 cases) were related to *Maekmundong-tang* [9]. According to a randomized comparative clinical trial wherein *Saengmaek-san* (modified formula) was administered for 7 days to patients with postinfectious cough, no adverse events, such as fatigue, dry throat, or globus sensation, occurred in the *Saengmaek-san* group on follow-up after 3 months [37]. Therefore, based on these previous studies, we will prioritize the safety of participants by systematically monitoring adverse events after the administration of *Maekmundong-tang* and *Saengmaek-san*, and will evaluate their safety profiles. If an adverse event occurs during the clinical trial period, it will be followed up by the researcher until it disappears.

The *Maekmundong-tang* and *Saengmaek-san* used in this study differed in the packaging amount of 2.5 and 1.41 g per serving, respectively. The composition and dosage of the two herbal medicines differ, and it was not possible to manufacture them with the same dosage due to the conditions applicable on the supplying pharmaceutical company. Therefore, we will try to keep the investigators and participants blinded during the trial period by packaging the two herbal medicines using the same opaque packaging.

## 4. Conclusions

In conclusion, we will explore the feasibility of future large-scale clinical trials and provide clinical evidence that can be immediately applied to the clinical setting by exploring the symptom improvement effect of *Maekmundong-tang* in patients with nonspecific chronic cough. In addition, the results of this study are expected to provide scientific evidence for the expansion of the health insurance coverage for chronic cough by comparing the cost-effectiveness of *Maekmundong-tang* with that of *Saengmaek-san*, which is an insurance-covered herbal medicine for cough. 

## Figures and Tables

**Table 1:** Schedule of enrolment, interventions, and assessments.

	Study Period					
	Enrolment	Allocation	Medication		Follow-Up	
Procedures	Visit 1 (Screening)	Visit 2	Visit 3	Visit 4	Visit 5	Visit 6 (Telephonic survey)
Timepoint	−3 to −1 weeks	Week 0	Week 3 ± 4 days	Week 6 ± 4 days	Week 9 ± 4 days	Week 24 ± 4 days
**ENROLLMENT**						
Eligibility screen	●					
Informed consent	●					
Demographics	●					
Medical and treatment history	●					
Physical examination *	●					
Chest X-ray, ECG, Pulmonary function test **	●					
Vital signs	●	●	●	●	●	
PICCQ		●				
Allocation		●				
**INTERVENTIONS**						
*Maekmundong-tang* or *Saengmaek-san*		●	●	●		
Multidimensional behavioral therapy guideline education		●	●	●	●	
**ASSESSMENTS**						
CSS ***		●	●	●	●	●
Cough VAS		●	●	●	●	●
LCQ		●	●	●	●	
HARQ		●	●	●	●	
IMOS/IMPSS			●	●	●	
EQ-5D-5L/EQ-VAS		●	●	●	●	●
Yin Deficiency Questionnaire		●		●		
Cost questionnaire ****		●	●	●	●	●
Laboratory tests *****	●			●		
Adverse events investigation		●	●	●	●	
Compliance test			●	●		

* Including auscultation as well as nasal and throat examinations; ** Including X-ray examination of paranasal sinuses, if considered necessary by the investigator; *** Assessed by cough diary, except for the telephone survey conducted at Week 24; **** Questionnaire to record medical, non-medical (transportation, time, and nursing care), and productivity-loss costs due to chronic cough of participants; ***** Including hematological parameters, fasting blood glucose level, liver and renal function tests, electrolyte test, total IgE, and fibrinogen level. The urinary human chorionic gonadotropin level was assessed at the screening visit for only women of childbearing age. Abbreviations: CSS, Cough Symptom Score; ECG, electrocardiogram; EQ-VAS, the EuroQol Visual Analog Scale; EQ-5D-5L, a five-level version of the EuroQol Five-dimensional Descriptive System; HARQ, Hull Airway Reflux Questionnaire; IMOS, Integrative Medicine Outcome Scale; IMPSS, Integrative Medicine Patient Satisfaction Scale; LCQ, Leicester Cough Questionnaire; PICCQ, Pattern Identification for Chronic Cough Questionnaire; VAS, visual analog scale.

## Data Availability

No datasets were generated or analyzed during the current study. All relevant data from this study will be made available upon study completion.
